# Quantifying mRNA in Highly Degraded Fixed Tissues by Nanostring Technology: A Comparative Study

**DOI:** 10.3390/mps7030040

**Published:** 2024-05-07

**Authors:** Eros Azzalini, Barbara Di Stefano, Vincenzo Canzonieri, Tiziana Venesio, Umberto Miglio, Caterina Marchiò, Anna Sapino, Carlo Previderè, Paolo Fattorini, Serena Bonin

**Affiliations:** 1 Department of Medical Sciences, University of Trieste, 34149 Trieste, Italy; eazzalini@units.it (E.A.); barbara.distefano@phd.units.it (B.D.S.); vcanzonieri@units.it (V.C.); sbonin@units.it (S.B.); 2 Pathology Unit, Centro di Riferimento Oncologico (CRO), IRCCS, Aviano-National Cancer Institute, 33081 Pordenone, Italy; 3 Candiolo Cancer Institute, Fondazione del Piemonte per l’Oncologia-IRCCS, 10060 Candiolo, Italy; tiziana.venesio@ircc.it (T.V.); umberto.miglio@ircc.it (U.M.); caterina.marchio@unito.it (C.M.); anna.sapino@unito.it (A.S.); 4 Department of Medical Sciences, University of Turin, 10126 Turin, Italy; 5 Laboratorio di Genetica Forense, Dipartimento di Sanità Pubblica, Medicina Sperimentale e Forense, Università di Pavia, 27100 Pavia, Italy; previdere@unipv.it

**Keywords:** nCounter, RNA, RNA degradation, FFPE, Bouin, RT-qPCR, RT-ddPCR

## Abstract

Archive tissues are the most available source of human tissues useful for molecular analysis in translational research. The main issues for those specimens are the modification and degradation of biomolecules, namely proteins, DNA, and RNA. In the last decade, several high-throughput analytical methods have been applied to archive tissues. Although histological tissues are fixed in neutral-buffered formalin nowadays, in the recent past, Bouin’s solution was also used in tissue processing. The present study aims to investigate the feasibility of nCounter Nanostring hybridization in quantifying mRNA in highly degraded samples, such as Bouin’s fixed and paraffin-embedded (BFPE) tissues, in comparison to the standard formalin-fixed and paraffin-embedded (FFPE) tissues as a source of RNA. A total of 16 paraffin-embedded tissue blocks from eight patients were analyzed (8 were FFPE and 8 were BEPE). Nanostring technology was applied to 300 ng of each RNA sample, whereas 360 ng of the same templates were retrotranscribed and submitted to qPCR and ddPCR. Our results show that the Nanostring technology outperforms the reference methods (ddPCR and qPCR) in detecting target mRNA in FFPE and BFPE samples. However, even Nanostring technology does not escape the limitation imposed by the degradation of the RNA templates, which could lead to misleading conclusions on the gene expression level.

## 1. Introduction

Archive tissues are the most available source of human tissues. These types of tissues, linked to the clinical information on patients’ history and outcome, are a valuable resource for molecular analysis in translational research, particularly for validating prognostic and predictive biomarkers in retrospective studies. Neutral-buffered formalin (NBF) is the fixative of choice in pathology. Still, other fixatives were used for particular purposes or cultural/historical reasons, such as Bouin’s solution (BS) in French-speaking countries [1]. For historical reasons, BS has been preferred over formalin in diagnostics for its better preservation of some morphological features, especially for biopsies requiring excellent nuclear details, such as testis and lymphoma [2].

BS fixative is a mixture of formalin, picric acid, and acetic acid, which exerts prolonged damage to tissue components, altering nucleic acids and proteins. Formalin leads to the crosslinks between proteins and between proteins and nucleic acids through the formation of methylene bridges; picric acid, instead, causes protein coagulation, while acetic acid allows a faster penetration of the other two compounds within the tissue [3]. Consequently, nucleic acids and proteins are hardly analyzed, since isolated biomolecules for molecular analysis are usually scarce and/or severely degraded. Moreover, several in situ techniques, such as immunohistochemistry, can give uncertain staining results on Bouin’s material, especially for nuclear biomarkers [4].

In the past decades, several studies have shown that BS fixed samples are amenable for nucleic acids and proteins analyses but only to a certain extent [2,3,5]. In recent years, new cutting edge technologies have allowed the assessment of mRNA expression from archive tissues with high sensitivity and high throughput levels, overcoming several drawbacks and limitations linked to using fixed specimens. One of these technologies is the Nanostring nCounter. This gene expression profiler can directly assess up to 770 genes simultaneously using multiplexed color-coded probes without requiring the reverse transcription step of RNA into cDNA. During the past years, this platform has gradually entered clinical settings, so several Nanostring assays have been developed for in vitro diagnostics [6]. 

Since disease-related molecules are locked in collections of Bouin-fixed and paraffin-embedded (BFPE) specimens located in several hospitals and cancer institutes, it is crucial to evaluate the performance of Nanostring nCounter in biomolecules obtained from those types of tissues and compare them with the gold standard methods such as reverse transcription (RT) quantitative PCR (qPCR) or the more sensitive droplet digital PCR (ddPCR). Furthermore, due to the extensive degradation of nucleic acids in Bouin’s material, these samples could be used as models for evaluating the molecular results from autoptic tissues or forensic samples [7], which usually show high levels of RNA degradation. In the present study, we have performed matched comparison analyses on ovarian cancer specimens, fixed both in formalin and BS, to assess the suitability of Bouin’s fixed tissues for mRNA analyses on Nanostring nCounter. We investigated the impact of isolation procedures on the RNA quantity and quality, and then, we validated the Nanostring results using RT-PCR and RT-ddPCR, determining the differences and the reliability of those three approaches in mRNA analyses.

## 2. Materials and Methods

### 2.1. Samples

Sixteen paraffin-embedded tissue blocks from 8 patients were collected at the National Cancer Institute CRO in Aviano (Italy). For each patient, two matched tissue blocks of the same surgical specimen were retrieved, one fixed in formalin and one in Bouin’s solution, for 8 matched pairs. All cases were obtained from debulking surgeries of high-grade serous ovarian cancer (HGSOC), carried out from 2002 to 2009, of the same grade and stage (G3 and FIGO stage IIIC). HGSOC cases have been selected for the HERCULES project funded by the European Union’s Horizon 2020 research and innovation program under grant agreement No. 667403. Fixation and embedding procedures were routinely performed in the laboratory during the patient’s surgery. The case series has already been used in previous studies [3,8].

To avoid biases in the molecular analyses, the hematoxylin and eosin (H&E) slides of each matched pair were evaluated to certify the same histotype, morphological pattern, and tumor content in matched samples. From each paraffin-embedded block, eight 10 μm thick sections were cut on a standard microtome, placed in two 1.5 mL microcentrifuge tubes, and stored at −80 °C until extraction.

### 2.2. RNA Isolation

Based on the quantitative and qualitative results obtained in optimizing the extraction protocol, the Maxwell^®^ RSC RNA FFPE kit (Cat. No. AS1440, Promega, Madison, WI 53711-5399, USA) was chosen for RNA purification in a final volume of 50 µL. One 10 μm thick section was processed, as recommended by the manufacturer. To prevent repetitive freezing and thawing cycles, the samples were subdivided into 8 µL aliquots and stored at −80 °C.

### 2.3. Quantitative and Qualitative mRNA Assessment

#### 2.3.1. Yield and Purity

The RNA concentration and purity were measured in duplicate by a Nanodrop ND 1000 spectrophotometer (Thermo Fischer Scientific, Waltham, MA 02451, USA) using 1 µL of each RNA solution. The A280/260 and A260/230 absorbance ratios were used to assess purity, considering a ratio between 1.8 and 2.0 to be pure.

#### 2.3.2. Integrity

The RNA integrity and the extent of RNA fragmentation were estimated by microcapillary electrophoresis in the Agilent 2100 BioAnalyzer using the RNA 6000 Nano kit (Agilent Technologies, Santa Clara, CA 95051, USA) following the manufacturer’s instructions. The RNA smear analysis, included in the Agilent BioAnalyzer 2100 Expert software 2.6, was used to analyze the fragments’ size distribution of RNA. The range of the fragments’ size analysis was manually set between 1 and 600 bp and divided into five smear regions (1–59, 60–149, 150–299, 300–449, and 450–600 nucleotides).

### 2.4. Reverse Transcription

Reverse transcription (RT) was carried out as already described [9]. Briefly, 360 ng of RNA (as assessed by Nanodrop) were primed with random hexamers and reverse-transcribed into cDNA in a 20 µL final volume using 250 U of M-MLV (Thermo Fischer Scientific, Waltham, MA 02451, USA; Cat. No. 28025013). The RT was carried out at 37 °C for 60 min; afterward, the enzyme was inactivated by heating at 70 °C for 10 min. Samples were aliquoted and stored at −80 °C until use.

#### Measurement of RT Efficiency

RNA and cDNA from matched formalin/Bouin’s samples were used to evaluate the efficiency of reverse transcription using the QuantiFluor^®^ RNA and ssDNA Systems (Cat. No. E3310 and E3190, respectively, Promega, Madison, WI 53711-5399, USA). Following the manufacturer’s instructions, the low standard calibration protocol was used for both assays. Two aliquots of the RT mixture without RNA were used as ‘blank samples’ for RNA and cDNA quantification (one aliquot underwent RT, whereas the second was used as a pre-RT control). To create ‘standard samples’, two additional aliquots of the RT mixture with no template RNA were used (one aliquot underwent RT, whereas the second was used as a pre-RT control); after that, they were combined with 1 ng of the 1:100 QuantiFluor^®^ RNA or ssDNA standard (provided with the kit). Ten microliters of the ‘blank’ and ‘standard’ samples were then mixed with two hundred microliters of the working solution containing 1:2000 QuantiFluor^®^ RNA or ssDNA dye to calibrate the instrument. Following calibration, 10 µL of each sample were measured in 200 µL of working solution to extrapolate the concentration of RNA and cDNA (ng/µL) from the relative fluorescence.

### 2.5. Nanotring

A Nanostring platform (nanoString, Seattle, WA 98109, USA) was used to analyze the RNA of the eight matched formalin/Bouin pairs. Briefly, 300 ng of input RNA (as assessed by Nanodrop) was hybridized to a Nanostring 48 plex Custom Probes Codeset (nanoString, Seattle, WA 98109, USA), consisting of 71 target sequences, at 65 °C for 18 h. The complete list of genes and probes of the Nanostring Codeset is based on the study of Chen et al. [10] and is reported in Supplementary Table S1.

The hybridization products were then processed at the nCounter Prep Station (nanoString, Seattle, WA 98109, USA) to wash off unbound capture and reporter probes and immobilize the hybridized complexes on a streptavidin-coated cartridge. Barcoded signals were acquired using the nCounter Digital Analyzer (nanoString, Seattle, WA 98109, USA), and the data were analyzed using the Nanostring software nSolver 4.0 (nanoString, Seattle, WA 98109, USA).

To correct the biases generated by assay efficiency, such as hybridization, purification, or binding, the data were normalized to exogenous positive controls and to the internal levels of 11 housekeeping (or reference) genes (ACTB, GAPDH, GUSB, HPRT1, RPL19, PPIA, TBP, B2M, HMBS, CALM2, and PGK1) using the software default settings.

For probes targeting different regions of the same RNA transcript, the expression values were averaged.

### 2.6. Quantitative PCR

Quantitative PCR (qPCR) assays were carried out on Mastercycler^®^ ep Realplex (Eppendorf, Hamburg, Germany). Amplification was made using JumpStart^TM^ Taq ReadyMix^TM^ (Sigma-Aldrich^®^, St. Louis, MO 63103, USA; Cat. No. D7440) and a mix of unlabeled PCR primers together with TaqMan probes for the following target genes: glyceraldehyde-3-phosphate dehydrogenase (GAPDH); β actin (ACTB); Hypoxanthine Phosphoribosyltransferase 1 (HPRT1); AKT Serine/Threonine Kinase 1, 2, and 3 (AKT1, AKT2, and AKT3); and Kruppel-like factor 16 (KLF16).

A summary of the primer sequences and amplicon lengths is reported in Supplementary Table S2.

The qPCR reactions for GAPDH, HPRT1, the AKT isoforms, KLF16, and ACTB were carried out in a 21 µL final volume. Every reaction included 2 µL of RT mixture (corresponding nominally to 24 ng of cDNA), 15 pmol of each primer, 4.5 mM MgCl_2_, and 4.2 pmol of TaqMan probe. All reactions were run in triplicate. No template controls (NTCs) were used in any run.

### 2.7. Droplet Digital PCR

The expression of ACTB (95 bp), GAPDH (100 bp), HPRT1 (91 bp), AKT1 (77 bp), AKT2 (71 bp), AKT3 (77 bp), and KLF16 (77 bp) were assessed by droplet digital PCR (ddPCR) (Bio-Rad, Hercules, CA 94547, USA) according to the manufacturer’s instructions. A total of 1.1 µL of RT mixture (corresponding nominally to 13.2 ng of cDNA) was used in each reaction. Reactions were performed in a 20 µL final volume using 1× ddPCR Supermix for Probes (no dUTP, Bio-Rad, Hercules, CA 94547, USA; Cat. No. 1863024); 18 pmol of GAPDH, ACTB, HPRT1, AKT1, AKT2, AKT3, and KLF16 primer each; and 5 pmol of TaqMan probe . NTCs were processed in each run. Droplet generation was performed in a QX200^TM^ Droplet Generator (Bio-Rad, Hercules, CA 94547, USA). Droplets’ emulsion was transferred to a 96-well semi-skirted plate (Bio-Rad, Hercules, CA 94547, USA; Cat. No. 12001925), heat-sealed at 175 °C, and PCR was run in a thermocycler (iCycler, Bio-Rad, USA) as follows: denaturation at 95 °C for 10 min; 40 cycles at 94 °C for 30 s and 54 °C (KLF16), 56 °C (GAPDH), or 58 °C (ACTB; HPRT1; and AKT1, AKT2, and AKT3) for 1 min; and enzyme inactivation at 98 °C for 10 min. After amplification, the droplets’ fluorescence was read in the QX200^TM^ Droplet Reader (Bio-Rad, Hercules, CA 94547, USA). Data from ddPCR were analyzed using QuantaSoft^TM^ software 1.7.4 , and the droplets’ count was fitted to a Poisson distribution to obtain the absolute concentration (copies/µL) of the target sequence.

### 2.8. Data Validation and Normalization Procedures

The raw data obtained from Nanostring and ddPCR were normalized according to the gene expression data analysis guidelines of nSolver (https://www.nanostring.com, accessed on 6 February 2024). The data obtained from qPCR were normalized using the 2^^∆∆Ct^ method [11]. Normalization for all three methods was performed using three housekeeping (HSK) genes, namely ACTB, GAPDH, and HPRT1.

As the absolute counts obtained from Nanostring and ddPCR could not be directly compared with the Ct values obtained by qPCR, the data from the two platforms were transformed into Cq equivalents, as described by Radke et al. [12], and then normalized using the 2^^∆∆Ct^ method. 

### 2.9. Statistical Analyses

Data distribution was assessed using the D’Agostino–Pearson and Shapiro–Wilk tests. The differential expression in formalin and Bouin’s tissues was evaluated by multiple *t*-test analysis; the Holm–Sidak method was used to correct the p-value for multiple comparisons. Pairwise and sample-wise correlation analyses were carried out using the Pearson or Spearman test according to data distribution. In the sample-wise analysis, samples were clustered by the hierarchical correlation clustering method. All *p*-values were calculated as two-sided, and values < 0.05 were considered statistically significant. Statistical analyses were performed using GraphPad Prism 8.2.1 (GraphPad Software, La Jolla, CA, USA) and R ver 4.3.0.

## 3. Results

### 3.1. Quantitative and Qualitative mRNA Assessment

After isolation by the Maxwell^®^ RSC RNA FFPE kit, the RNA obtained from eight matched samples fixed in formalin and BS was tested for quantity, quality, and integrity. The quantity of the RNA was similar in matched FFPE/BFPE tissues, but the RNA purity was significantly lower in the BFPE samples compared to the FFPE ones for both A260/280 (*p* < 0.0001) and A260/230 (*p* = 0.04) ratios. Although the RIN numbers were comparable between RNA obtained from the two fixatives, the smear analysis revealed more RNA fragments of lengths between 150 and 299 nucleotides in the formalin samples compared to Bouin’s (*p* = 0.01), indicating better preservation in the FFPE samples. The results of the isolation procedure are reported in Supplementary Figures S1 and S2.

### 3.2. Nanostring Analysis

**Figure 1:**
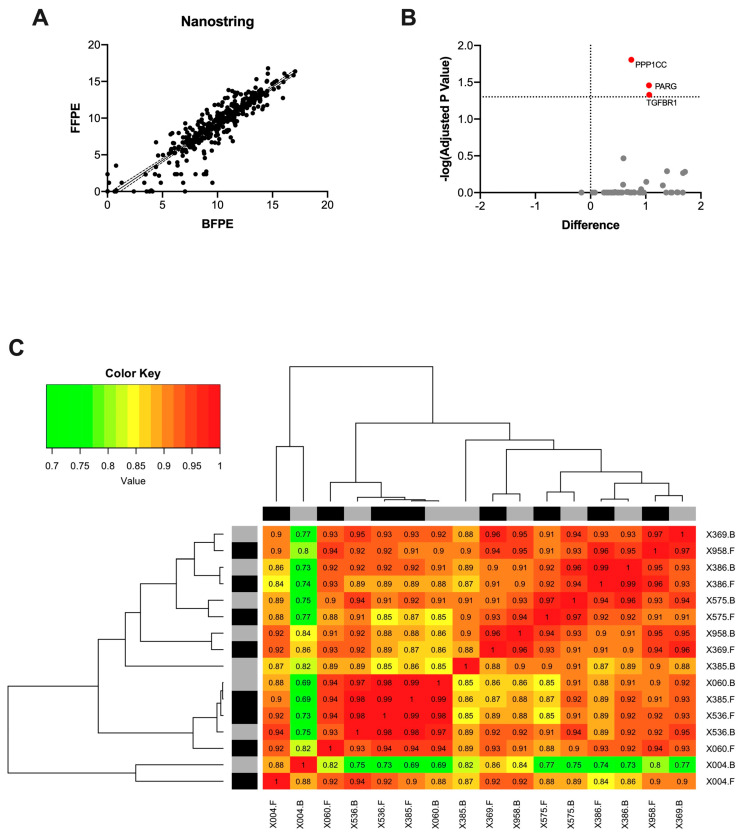
Pairwise correlation of the normalized transcript abundance for 49 target genes in formalin and Bouin’s tissues as detected by Nanostring. The mean pairwise correlation was 0.91 (*p* < 0.0001) (**A**). Volcano plot for differentially expressed genes on matched formalin and Bouin’s tissues identified on Nanostring (**B**). Heatmap with hierarchical clustering analysis for the sample-wise correlations of transcript abundance detected by Nanostring. Black and grey bars indicate FFPE and BFPE samples, respectively (**C**).

The expression of 71 mRNA transcripts, including 11 housekeeping genes and 49 target genes, was analyzed in the eight formalin/Bouin’s matched pairs using Nanostring nCounter^®^. The geNORM algorithm was employed to determine the best housekeeping gene for normalization. Based on this analysis, the B2M gene was discarded because of its limited stability; therefore, the data were normalized using the remaining 10 housekeeping genes. The raw data obtained by Nanostring was log2 transformed, and the normalized quantification values derived from the eight paired FFPE and BFPE samples were compared. The pair-wise Pearson correlation coefficient was 0.91 (*p* < 0.0001). The scatter plot displaying log2 values from the formalin and Bouin’s samples is presented in Figure 1A.

A sample-wise correlation was also conducted to determine whether transcript abundance was conserved in each sample pair. The results indicated that the gene expression patterns were highly consistent across all samples, with a mean correlation coefficient of 0.94 (ranging from 0.86 to 0.99). Only two samples (samples X004 and X385) exhibited correlation values lower than 0.94 (Pearson coefficient = 0.88 and 0.86, respectively). Figure 1C shows the unsupervised hierarchical clustering and heatmap analysis of the sample-wise correlations. In the matched formalin/Bouin’s tissues, multiple *t*-test comparisons revealed that 10 out of 49 target genes (TGFBR1, PARG, PPP1CC, AKT1, RP11-247A12.2.1, TFEC, BEST1, CXCL3, MAP1S, and AKT3) had decreased expression in the BFPE samples. However, after adjusting the *p*-value for multiple comparisons, only three genes, namely TGFBR1, PARG, and PPP1CC, remained significantly different (Figure 1B).

### 3.3. Data Validation

The Nanostring results of the formalin and Bouin’s tissues were validated using seven selected targets by ddPCR and qPCR. The transcripts levels of three HSK genes (GAPDH, ACTB, and HPRT1) used for data normalization; three gene isoforms at different levels of expression (AKT1, AKT2, and AKT3) and one gene at a very low level of expression (KLF16) were selected and compared.

#### 3.3.1. Correlation between FFPE and BFPE Tissues through Nanostring

**Figure 2:**
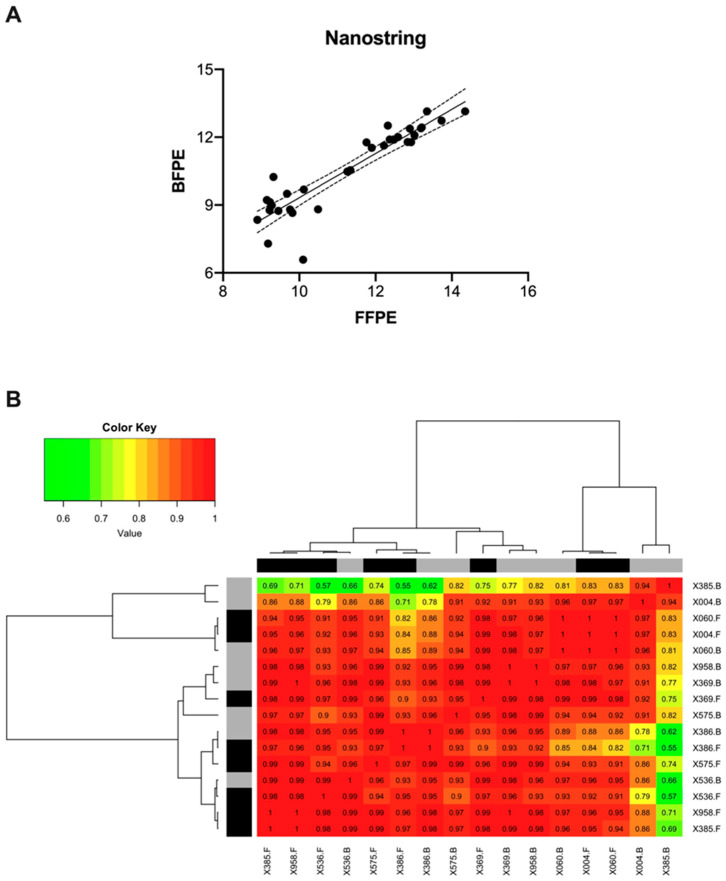
Pairwise correlation of normalized transcript abundance for the AKT1, AKT2, AKT3, and KLF16 target genes in formalin and Bouin’s tissues as detected by Nanostring. The mean pairwise correlation was 0.95 (*p* < 0.0001) (**A**). Heatmap with hierarchical clustering analysis for the sample-wise correlations of transcript abundance as detected by Nanostring. Black and grey bars indicate FFPE and BFPE samples, respectively (**B**).

Nanostring raw data was extracted and log2-transformed. The normalized expression values of AKT1, AKT2, AKT3, and KLF16 were then compared in matched FFPE and BFPE tissues. The Pearson pairwise correlation was 0.91 (*p* < 0.0001). The scatter plot displaying log2 values from the formalin and Bouin’s samples is presented in Figure 2A. A sample-by-sample comparison of transcript abundance between matched formalin/Bouin’s pairs was also conducted, and the results showed a mean correlation of 0.95, with a minimum of 0.69 and a maximum of 1. Notably, seven out of eight samples had correlation values ≥ 0.97, whereas sample X385 showed a minimum value of 0.69. Figure 2B shows the corresponding heatmap with unsupervised hierarchical clustering of the data correlations.

#### 3.3.2. Correlation between FFPE and BFPE Tissues through RT-ddPCR

**Figure 3:**
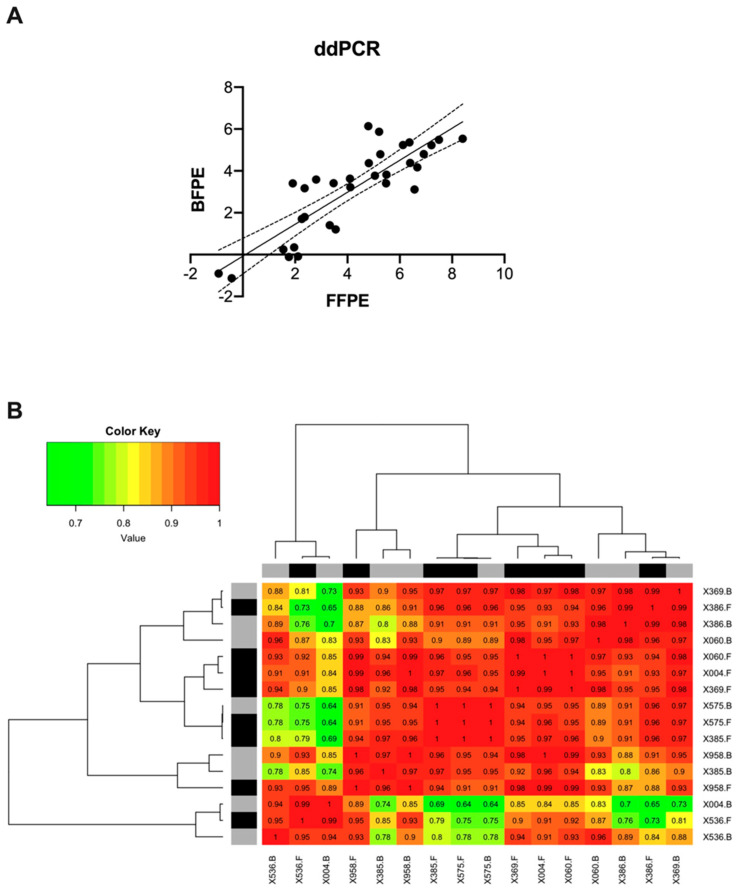
Pairwise correlation of transcript abundance for the AKT1, AKT2, AKT3, and KLF16 genes in formalin and Bouin’s tissues as detected by ddPCR. The mean pairwise correlation was 0.86 (*p* < 0.0001) (**A**). Heatmap with hierarchical clustering analysis for the sample-wise correlations of transcript abundance as detected by ddPCR. Black and grey bars indicate FFPE and BFPE samples, respectively (**B**).

The gene expression levels of the selected targets were compared in FFPE and BFPE samples using droplet digital PCR. The pairwise Pearson coefficient was 0.85 (*p* < 0.0001) (see Figure 3A). A sample-wise correlation analysis showed a mean correlation of 0.96, ranging from 0.84 to 1. Similar to Nanostring, seven out of eight samples exhibited correlation coefficients of ≥0.95. In this case, sample X004 had the lowest correlation value of 0.88. The results are presented in a heatmap and hierarchical clustering in Figure 3B.

#### 3.3.3. Correlation between FFPE and BFPE Tissues through qPCR

**Figure 4:**
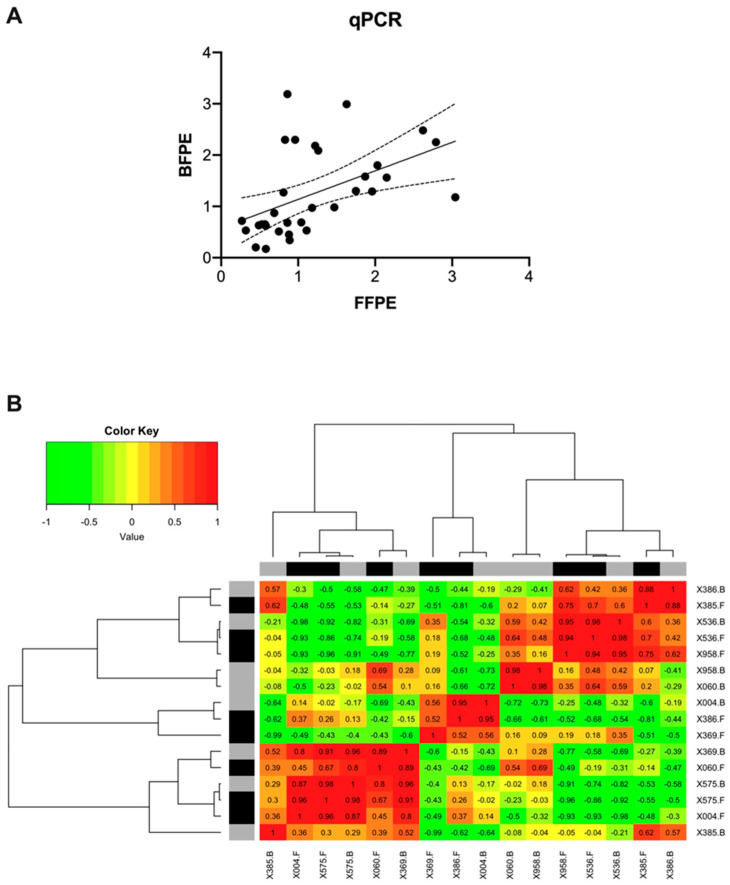
Pairwise correlation of normalized transcript abundance for 4 target genes (AKT1, AKT2, AKT3, and KLF16) in formalin and Bouin’s tissues as detected by qPCR. The mean pairwise correlation was 0.61 (*p* = 0.0002) (**A**). Heatmap with hierarchical clustering analysis for the sample-wise correlations of transcript abundance as detected by qPCR. Black and grey bars indicate FFPE and BFPE samples, respectively (**B**).

Matched tissue comparisons were also performed for qPCR. The mean pairwise correlation of the data was 0.61 (*p* < 0.0001), as depicted in Figure 4A. A sample-by-sample comparison returned a mean coefficient of 0.30 with a minimum of −0.60 and a maximum of 0.98. In this case, only two samples, X536 and X575, exhibited correlation coefficients ≥0.95. Strikingly, two samples (X386 and X369) were completely uncorrelated, exhibiting negative correlation values of −0.44 and −0.60, respectively. The respective heatmaps are presented in Figure 4B.

#### 3.3.4. Differential Expression in FFPE and BFPE Tissues

**Figure 5:**
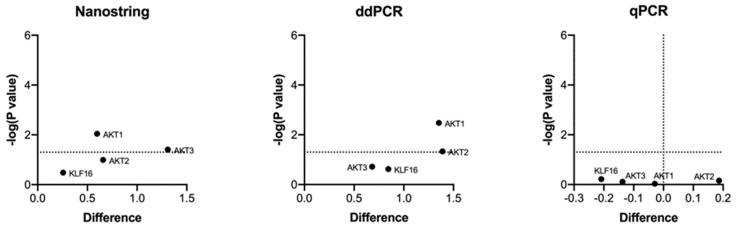
Volcano plot for differentially expressed genes on matched formalin and Bouin’s tissues identified on Nanostring, ddPCR, and qPCR using log2 normalized data. Dotted lines represent the threshold for *p*-value significance (*p* < 0.05).

The expression levels of each gene in the FFPE and BFPE specimens were compared using the Nanostring, ddPCR, and qPCR platforms. A multiple *t*-test analysis revealed no differential expression between the two fixatives in qPCR, while two targets showed different expression on the Nanostring and ddPCR platforms. The results are presented in a volcano plot (see Figure 5).

### 3.4. Comparison of Gene Expression Values Using Nanostring, ddPCR, and qPCR

The gene expression levels were compared among Nanostring, RT-ddPCR, and qPCR. The data obtained from Nanostring and ddPCR were directly compared for each type of fixative, as both platforms rely on absolute counts. To facilitate the comparison between absolute counts and the Ct values obtained from qPCR, instead, the Nanostring and ddPCR data were converted into Ct equivalents, as described in the Section 2.

**Figure 6:**
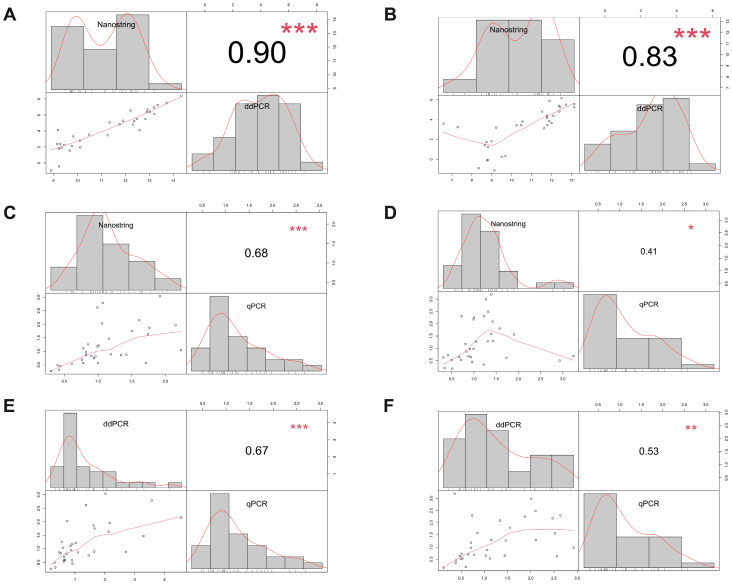
Scatterplot matrix representing the pairwise correlation of transcript abundance between Nanostring, ddPCR, and qPCR in FFPE (**A**,**C**,**E**) and BFPE (**B**,**D**,**F**) tissues. ***: *p*-value < 0.0001; **: *p*-value < 0.001; *: *p*-value < 0.01.

In the FFPE tissues, the pairwise correlation coefficient between Nanostring and ddPCR was 0.90 (*p* < 0.0001), while, between Nanostring and qPCR, it was 0.68 (*p* < 0.0001). The mean Spearman correlation coefficient between ddPCR and qPCR was 0.67 (*p* < 0.0001). The same analysis performed on the Bouin’s tissues revealed lower correlation values. The mean level of concordance between Nanostring and ddPCR was 0.83 (*p* < 0.0001), while, between Nanostring and qPCR, it was 0.41 (*p* = 0.02). The correlation between ddPCR and qPCR was 0.53 (*p* = 0.002). Figure 6A–F shows the scatterplot matrices of inter-platform correlations in the FFPE and BFPE specimens. A sample-by-sample correlation analysis was also carried out among the three platforms. In the FFPE tissues, the mean sample correlation between Nanostring and ddPCR was 0.94 (range 0.88–1) and between Nanostring and qPCR was 0.69 (range 0.30–0.91), while, for ddPCR vs. qPCR, it was 0.61 (range −0.41 to 0.97). Overall, the correlations between samples were weaker for the Bouin’s fixed material. The Pearson coefficient was 0.85 (range 0.61–0.96) for Nanostring vs. ddPCR, 0.18 (range −0.88 to 0.85) for Nanostring vs. qPCR, and 0.36 (range −0.64 to −0.92) for ddPCR vs. qPCR.

### 3.5. Measurement of RT Efficiency

**Figure 7:**
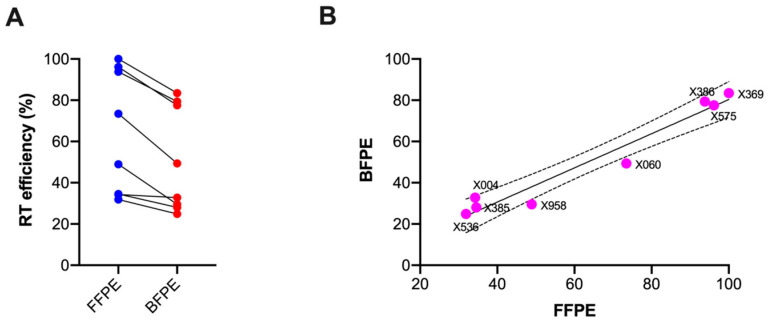
Reverse transcription efficiency in matched formalin and Bouin’s tissues (**A**), and the respective correlation in the two groups (**B**).

The results obtained both from RT-qPCR and RT-ddPCR showed lower levels of agreement between the formalin and Bouin’s tissues if compared to Nanostring. Moreover, in RT-qPCR and RT-ddPCR, the raw expression levels of five out of seven genes significantly differed between the formalin and Bouin’s tissues. In contrast, the Nanostring analysis revealed that only two out of seven genes were differentially expressed (see Supplementary Figure S3). To investigate whether this discrepancy could be attributed to inefficient cDNA synthesis, the amount of RNA and the corresponding cDNA generated by reverse transcription were measured in matched sample pairs using QuantiFluor^®^ RNA and ssDNA Systems. The results indicated a lower efficiency of reverse transcription in the Bouin’s fixed samples compared to the formalin-fixed samples (*p* = 0.002) (Figure 7A). The mean RT efficiency in the FFPE tissues was 64% (range 32–100%), while, in Bouin’s, it was 51% (range 25–83%), in agreement with higher levels of template damage in the BFPE samples [5,6]. However, a strong linear correlation was found between the RT efficiencies from the matched samples (R^2^ = 0.97; *p* < 0.0001) (Figure 7B). Notably, the correlation between the RT efficiency and A260/280 ratio was similar in the FFPE and BFPE samples (r = 0.72 and 0.80, respectively), while the correlation between the RT efficiency and 260/230 was higher in the Bouin’s-fixed tissues compared to the formalin-fixed tissues (r = 0.52 vs. 0.27), likely due to the presence of the aromatic ring of picric acid in the Bouin’s fixative.

## 4. Discussion

In this study, we used three different technologies (Nanostring, RT-ddPCR, and RT-qPCR) to measure the gene expression in matched FFPE and BFPE samples of high-grade serous ovarian cancer. Overall, nucleic acids from the Bouin’s tissues were more degraded compared to formalin, as shown by RNA fragment analysis on the Agilent BioAnalyzer. However, despite the higher degradation, they were still accessible for gene expression analysis on all three platforms.

Other authors, including our group, have reported that Bouin’s tissues are suitable for mRNA analysis by RT-qPCR but only up to a certain amplicon length due to extensive degradation [2,6]. Golghini et al. [5] found a very strong correlation between matched fresh, FFPE, and BFPE lymphoid tissues by qPCR analysis, but the fixation time was controlled to 4 h, which is far from the time of fixation in the routine (usually 24 h). To our knowledge, there are no reports on the performance of Nanostring to analyze the abundance of mRNA transcripts on BFPE material fixed under standard conditions.

Our results showed that the expression values obtained from formalin and Bouin’s samples were very highly correlated on the Nanostring platform, even when analyzing up to 71 transcripts. Data validation using selected targets confirmed that FFPE/BFPE matched tissues had the highest level of correlation on the Nanostring platform, followed by ddPCR and qPCR. In addition, the sample-wise analysis showed a very high level of concordance between Nanostring and ddPCR, whereas qPCR exhibited high variability, with two samples having completely uncorrelated values. Several factors may account for this discrepancy, including the different efficiency of reverse transcription between the formalin and Bouin’s tissues and the method of normalization. The presence of aromatic inhibitors in BFPE clearly affected both reverse transcription and qPCR, confirming previous reports [3,13,14]. With ddPCR, it was less evident, likely due to the resilience of the method toward inhibitors [14].

To date, there is no general consensus on the correct method for comparing normalized absolute counts derived from Nanostring and RT-ddPCR with relative quantification values obtained by RT-qPCR. In addition, as already reported [13], normalization can compensate only to a certain extent for nucleic acid degradation, while the performance of reference genes can vary between platforms. The use of synthetic standards and the results expressed as a relative change with respect to a reference sample are the most common approaches to facilitate comparisons [15], even if limitations are expected in highly degraded samples. Other methods, such as the one used in this study, involve the transformation of absolute counts into Ct values using a mathematical approach with several limitations. Nevertheless, in agreement with other authors, we obtained a moderate to good correlation when comparing the Nanostring and ddPCR results with qPCR in the FFPE samples [16,17]; as expected, for the BFPE tissues, the inter-platform correlations were poorer.

As mentioned above, differences in reverse transcription efficiency may also have contributed to the different expression levels and poorer inter-platform correlations in the BFPE samples compared to FFPE. Our results based on the QuantiFluor^®^ instrument clearly showed that the Bouin’s fixed tissues yielded, on average, 15% less cDNA than formalin, which could limit their analysis on RT-based platforms. In addition, sequence-specific damages could interfere with the RT efficiency in a nonrandom way. Even replicated RTs are recommended for assessing accurate expression levels [13]; still, limitations in sample availability represent a common issue in routine practice. Although expensive and time-consuming, this recommendation should be followed in future studies to better understand the outcomes of these degraded samples in different platforms.

Notably, although both ddPCR and qPCR share the same reverse transcription step, in our study, ddPCR showed a better overall performance compared to qPCR, also resulting in highly correlated data with Nanostring. This is in agreement with several authors, who have shown that ddPCR, due to its higher sensitivity and resilience to PCR inhibitors, may be a more suitable method compared to qPCR for analyzing highly degraded samples with varying levels of chemical and protein contaminants, such as those fixed in Bouin’s solution [18,19]. In conclusion, our results suggest that the nucleic acid quality can significantly affect the gene expression analysis, especially on RT-based platforms such as RT-ddPCR and RT-qPCR. On the other hand, hybridization methods such as Nanostring may be more reliable for gene expression analysis, especially in highly degraded samples such as Bouin’s fixed or autoptic tissues, with the advantage of a high multiplexing capability. Nevertheless, after normalization, three transcripts not apparently interconnected were expressed significantly lower in the BFPE tissues. Limitations of this platform include its high cost and the need for large amounts of input RNA (300 ng for degraded RNA, as reported by the manufacturer) to perform an analysis on fixed material, factors that may favor the use of the traditional PCR-based methods, such as ddPCR or qPCR. In this case, however, droplet digital PCR should be preferred to qPCR for gene expression analysis in these types of tissues due to its higher sensitivity and resilience to PCR inhibitors. Anyway, we acknowledge as the main limitation of this study the small sample size; therefore, more extensive studies are welcome to better understand the role of degraded/modified RNA molecules in the fidelity of the molecular assays performed in routine tests.

## Data Availability

The data not presented in this work are available upon request.

## Supplementary Materials

The following supporting information can be downloaded at https://www.mdpi.com/article/10.3390/mps7030040/s1: Table S1. Target gene elements present in the Nanostring Codeset; Table S2. Amplification primers used for ddPCR and qPCR analysis; Figure S1. Agilent BioAnalyzer profiles of matched FFPE/BFPE pairs and respective RIN numbers; Figure S2. Results of the isolation procedure in the eight matched formalin/Bouin’s samples. Figure S3. Volcano plot for differentially expressed genes on matched formalin and Bouin’s tissues identified on Nanostring, ddPCR and qPCR using raw data (C).
