# Supplementary material for: Quantifying mRNA in Highly Degraded Fixed Tissues by Nanostring Technology: A Comparative Study
Source: Methods Protoc. 2024 May 7;7(3):40. doi: 10.3390/mps7030040 (PMC11130824; doi:10.3390/mps7030040)

SUPPLEMENTARY MATERIAL

Table S1. Target gene elements present in the Nanostring CodeSet

Gene symbol	Accession number	Position	Target sequence
AC015971.2.1	ENST00000426549.1	305-404	CTCCGACATTTCCTCTTCAGACTGGACTCCAAGACATTTAAATCGCCACATTGAGACTATACGTAACATGGACCTGGAAAAGCTTAAAAATATGAAGAGG
AC083884.8	ENST00000434256.1	679-778	AGATGGTCCCATCCTGGCATATTCTCAGGTACAATTTGGCCTCAGAATACAGAAGCCCAGCACATCCAACTATTACCATAAAATACCAGGAATGTTTTGG
ACTB	NM_001101.2	1011-1110	TGCAGAAGGAGATCACTGCCCTGGCACCCAGCACAATGAAGATCAAGATCATTGCTCCTCCTGAGCGCAAGTACTCCGTGTGGATCGGCGGCTCCATCCT
ADAM12	NM_003474.4	1925-2024	CAACGTGTACCTGCACGATGGGCACTCATGTCAGGATGTGGACGGCTACTGCTACAATGGCATCTGCCAGACTCACGAGCAGCAGTGTGTCACGCTCTGG
ADAM12_b	NM_003474.5	639-738	AGCAAAGAACTGATCATAAATCTGGAAAGAAATGAAGGTCTCATTGCCAGCAGTTTCACGGAAACCCACTATCTGCAAGACGGTACTGATGTCTCCCTCG
ADAMTS12_b	NM_030955.2	1956-2055	CAGAGGCACCAACATTTCGGCAGATGCAGTGCAGTGAATTTGACACTGTTCCCTACAAGAATGAACTCTACCACTGGTTTCCCATTTTTAACCCAGCACA
ADAMTS12	XM_011514147.1	3549-3648	CTGGTTACATCCCTCAGTCTCTTCCTCCATCCCTGTAGGGCTCCATATCTGGGCTGCTTAGAGTCTCCTGCCAACCTTGGGACACTAGACTAAGAGGAAA
AEBP1_b	NM_001129.4	1464-1563	CCCATTGGGATGGAGTCACACCGTATTGAGGACAACCAGATCCGAGCCTCCTCCATGCTGCGCCACGGCCTGGGGGCACAGCGCGGCCGGCTCAACATGC
AEBP1_c	NM_001129.4	1724-1823	CTTCTTCGTGGGCTTCAGCAATGACAGCCAGACATGGGTGATGTACACCAACGGCTATGAGGAAATGACCTTTCATGGGAACGTGGACAAGGACACACCC
AEBP1	NM_001129.4	2560-2659	AGAAGAACCCCTTCGTGCTGGGAGCAAATCTGAACGGCGGCGAGCGGCTAGTATCCTACCCCTACGATATGGCCCGCACGCCTACCCAGGAGCAGCTGCT
AKT1	NM_001014432.1	1276-1375	GGACGGGCACATTAAGATCACAGACTTCGGGCTGTGCAAGGAGGGGATCAAGGACGGTGCCACCATGAAGACCTTTTGCGGCACACCTGAGTACCTGGCC
AKT2	NM_001626.4	700-799	AGGAGATGGAAGTGGCGGTCAGCAAGGCACGGGCTAAAGTGACCATGAATGACTTCGACTATCTCAAACTCCTTGGCAAGGGAACCTTTGGCAAAGTCAT
AKT3	NM_005465.4	288-387	GCCAGTTAATGAAAACAGAACGACCAAAGCCAAACACATTTATAATCAGATGTCTCCAGTGGACTACTGTTATAGAGAGAACATTTCATGTAGATACTCC
ATP1A1-AS1	NR_027646.1	122-221	CCTCGTGGGGTGTGTGAGGGCCGAGTGAAATCGTGCATTTGCGGTCTGAAATGCAGAAGACAGAAGATGAGCTCTGGCTCTTCACCTGCATGGCTGGTGG
B2M	NM_004048.2	236-335	TACTGAAGAATGGAGAGAGAATTGAAAAAGTGGAGCATTCAGACTTGTCTTTCAGCAAGGACTGGTCTTTCTATCTCTTGTACTACACTGAATTCACCCC
BEST1	NM_004183.3	1119-1218	TGTTCTGGGTGCCCTGGGTGTGGTTTGCCAACCTGTCAATGAAGGCGTGGCTTGGAGGTCGAATCCGGGACCCTATCCTGCTCCAGAGCCTGCTGAACGA
BICC1	NM_001080512.1	2156-2255	CACGGTCATCATATGTCAACATGCAGGCATTTGACTATGAACAGAAGAAGCTATTAGCCACCAAAGCTATGTTAAAGAAACCAGTGGTGACGGAGGTCAG
CALM2	NM_001743.3	869-968	TGGAGTTGTAACTCTGCGTGGACTATGGACAGTCAACAATATGTACTTAAAAGTTGCACTATTGCAAAACGGGTGTATTATCCAGGTACTCGTACACTAT
CD68	NM_001251.2	1141-1240	ACCGGTCCATCTTGCTGCCTCTCATCATCGGCCTGATCCTTCTTGGCCTCCTCGCCCTGGTGCTTATTGCTTTCTGCATCATCCGGAGACGCCCATCCGC
COL10A1	NM_000493.3	136-235	AACTTGGTTCATGGAGTGTTTTACGCTGAACGATACCAAATGCCCACAGGCATAAAAGGCCCACTACCCAACACCAAGACACAGTTCTTCATTCCCTACA
COL1A2	NM_000089.3	2636-2735	CCAATGGATTTGCTGGTCCTGCTGGTGCTGCTGGTCAACCTGGTGCTAAAGGAGAAAGAGGAGCCAAAGGGCCTAAGGGTGAAAACGGTGTTGTTGGTCC
COL3A1	NM_000090.3	181-280	TTGGCACAACAGGAAGCTGTTGAAGGAGGATGTTCCCATCTTG

			GTCAGTCCTATGCGGATAGAGATGTCTGGAAGCCAGAACCATGCCAAATATGTGTCT
COL3A1_b	NM_000090.3	2263-2362	CCTGGTGCTGCTGGTACTCCTGGTCTGCAAGGAATGCCTGGAGAAAGAGGAGGTCTTGGAAGTCCTGGTCCAAAGGGTGACAAGGGTGAACCAGGCGGTC
COL5A1_b	NM_000093.3	494-593	CAGCCAGCAGATCTCCTGAAGGTTCTAGATTTTCACAACTTGCCTGATGGAATAACAAAGACAACAGGCTTTTGCGCCACGCGGCGATCTTCCAAAGGCC
COL5A1	NM_000093.3	873-972	AGTGGCACAGAATTGCTCTCAGCGTCCACAAGAAAAATGTCACCTTGATCCTCGACTGTAAAAAGAAGACCACCAAATTCCTCGACCGCAGCGACCACCC
COL5A2	NM_000393.3	4076-4175	GGTTCATGCTACCCTGAAGTCACTCAGTAGTCAGATTGAAACCATGCGCAGCCCCGATGGCTCGAAAAAGCACCCAGCCCGCACGTGTGATGACCTAAAG
COL5A2_b	NM_000393.3	5761-5860	TGGAATTAGACCATTTGGCCTTTGAACTTTCATAGGAAAAATGACCCAACATTTCTTAGCATGAGCTACCTCATCTCTAGAAGCTGGGATGGACTTACTA
COL6A1_b	NM_001848.2	2303-2402	CTCAGAGGGACACCACACCGCTCAACGTGCTCTGCAGCCCCGGCATCCAGGTGGTCTCCGTGGGCATCAAAGACGTGTTTGACTTCATCCCAGGCTCAGA
COL6A2	NM_001849.2	1926-2025	TCGTCATCGACAGCTCCGAGAGCATTGGGTACACCAACTTCACACTGGAGAAGAACTTCGTCATCAACGTGGTCAACAGGCTGGGTGCCATCGCTAAGGA
COL8A1	NM_001850.3	2141-2240	AGTGAAGTTTAACAAACTGCTGTATAACGGCAGACAGAACTACAACCCGCAGACAGGCATCTTCACCTGTGAGGTCCCTGGTGTCTACTACTTTGCATAC
COLEC12	NM_130386.2	901-1000	ACACAAGCCAGGCTATCCAGCGAATCAAGAACGACTTTCAAAATCTGCAGCAGGTTTTTCTTCAAGCCAAGAAGGACACGGATTGGCTGAAGGAGAAAGT
CTSK	NM_000396.2	416-515	TCTCATTCCCGCAGTAATGACACCCTTTATATCCCAGAATGGGAAGGTAGAGCCCCAGACTCTGTCGACTATCGAAAGAAAGGATATGTTACTCCTGTCA
CXCL3	NM_002090.2	541-640	TCCCTGCCCTTACCAGAGCTGAAAATGAAAAAGAGAACAGCAGCTTTCTAGGGACAGCTGGAAAGGACTTAATGTGTTTGACTATTTCTTACGAGGGTTC
ECM1	NM_004425.3	1346-1445	TGTTGCCGCCACCCTCCCAGCCCTACTCGGGATGAGTGCTTTGCCCGTCGGGCTCCTTACCCCAACTATGACCGGGACATCTTGACCATTGACATCGGTC
FAP_b	NM_004460.2	1491-1590	GCATTGGAAGCTATCCTCCAAGCAAGAAGTGTGTTACTTGCCATCTAAGGAAAGAAAGGTGCCAATATTACACAGCAAGTTTCAGCGACTACGCCAAGTA
FN1	NM_212482.1	1777-1876	GGGAATGGACATGCATTGCCTACTCGCAGCTTCGAGATCAGTGCATTGTTGATGACATCACTTACAATGTGAACGACACATTCCACAAGCGTCATGAAGA
GAPDH	NM_001256799.1	387-486	GAACGGGAAGCTTGTCATCAATGGAAATCCCATCACCATCTTCCAGGAGCGAGATCCCTCCAAAATCAAGTGGGGCGATGCTGGCGCTGAGTACGTCGTG
GUSB	NM_000181.3	1900-1999	CCGATTTCATGACTGAACAGTCACCGACGAGAGTGCTGGGGAATAAAAAGGGGATCTTCACTCGGCAGAGACAACCAAAAAGTGCAGCGTTCCTTTTGCG
HMBS	NM_000190.3	316-415	CATTGCTATGTCCACCACAGGGGACAAGATTCTTGATACTGCACTCTCTAAGATTGGAGAGAAAAGCCTGTTTACCAAGGAGCTTGAACATGCCCTGGAG
HPRT1	NM_000194.1	241-340	TGTGATGAAGGAGATGGGAGGCCATCACATTGTAGCCCTCTGTGTGCTCAAGGGGGGCTATAAATTCTTTGCTGACCTGCTGGATTACATCAAAGCACTG
INTS5	NM_030628.1	3026-3125	CCTCCACCGCAACATCGACCGCCTAGGTCTTTTCTCTGGCCGTTTCCAGGCACCTTCACCGTCCACTCTCCTTCGACAGGGGACGTAGCCTTTTCTTGCT
INTS5_b	NM_030628.1	685-784	CCTTGCTGGATACCTCTGTTCAGCATTCTCCACACTTTGACTGGGTTGTGGCACATATTGGCTCCTCTTTTCCTGGCACCATCATTTCCCGGGTTCTCTC
ISM1	NM_080826.1	109-208	GCGGCCGCGGGCAACGCCAGCCAAGCCCAGCTGCAGAATAACCTCAACGTGGGAAGTGACACCACATCAGAAACCAGCTTTTCTCTCTCCAAAGAAGCAC
ITGA5_b	NM_002205.2	2629-2728	AAGGTCAGCAGCTCCTATATGTGACCAGAGTTACGGGACTCAACTGCACCACCAATCACCCCATTAACCCAAAGGGCCTGGAGTTGGATCCCGAGGGTTC
ITGA5	NM_002205.2	4114-4213	GCCAGCTGCACTGATGCTGCCCCTCATCTCTCTGCCCAACCCTTCCCTCACCTTGGCACCAGACACCCAGGACTTATTTAAACTCTGTTGCAAGTGCAAT
KDM4A-AS1	NR_033827.1	339-438	ATGGCTGAGAATCCCTTGGACCCTGGAAGCACCTACTCCATGATGGCCCGCAGGAGGACCAAACTGGAGACAGTCCTGGCTCTGA

			GGAAACAGGAAGGAA
KLF16	NM_031918.3	1521-1620	CTCTGGGTTCTTCCAAAGAACATTTGACTCAGTGGGGTCGGGAGGGAATCCTGGCTTATTTATTTTCCTCTGTCCTCGGGTGTGGGGGCCCTAGCTCGCC
KLF16_b	NM_031918.3	2298-2397	CCCCTTTTGTTCCAGAACCGTCCTGCCTCTCGCTTGGGGACAGGGGGGCTTTGGATGGCACTGGTGTGCACCTGGACCCAGCCCCGGCCTGGCAGGATCC
MAP1S_b	NM_018174.4	268-367	ACCCTGGTCCTCCTGAACCCATCAGACAAGTCCCTGTATGATGAGCTCCGGAACCTTCTGTTGGACCCTGCCTCTCACAAGCTACTGGTGTTGGCTGGGC
MMP11	NM_005940.3	703-802	AGCAGCCAAGGCCCTGATGTCCGCCTTCTACACCTTTCGCTACCCACTGAGTCTCAGCCCAGATGACTGCAGGGGCGTTCAACACCTATATGGCCAGCCC
MMP13	NM_002427.2	952-1051	CTGGCGCCTGCATCCTCAGCAGGTTGATGCGGAGCTGTTTTTAACGAAATCATTTTGGCCAGAACTTCCCAACCGTATTGATGCTGCATATGAGCACCCT
MMP14	NM_004995.2	1471-1570	GACAAGATTGATGCTGCTCTCTTCTGGATGCCCAATGGAAAGACCTACTTCTTCCGTGGAAACAAGTACTACCGTTTCAACGAAGAGCTCAGGGCAGTGG
MMP14_b	NM_004995.3	2733-2832	AAACCCTTGGCAGCCCTGTGCCTCTCGAATGTTAGCCTTGGATGGGGCTTTCACAGTTAGAAGAGCTGAAACCAGGGGTGCAGCTGTCAGGTAGGGTGGG
MMP2_b	NM_004530.5	1719-1818	AAACAGGACATTGTATTTGATGGCATCGCTCAGATCCGTGGTGAGATCTTCTTCTTCAAGGACCGGTTCATTTGGCGGACTGTGACGCCACGTGACAAGC
MYPOP	NM_001012643.3	300-399	CACGGGCCAGGAGGTGCAGAAGCGCTGGAACGACTTCAAGCGCCGCACCAAGGAGAAGCTCGCTCGCGTGCCGCACTCCACGCAGGGCGCCGGGCCCGCC
NCKAP5L	NM_001037806.3	337-436	GGCACTTGCCCAGGCCAACGAAAACCAGCGGGAGACTTATGAGCGCTGTCTGGACGAGGTTGCCAACCATGTGGTACAGGCGTTGCTGAACCAGAAGGAC
PARG	NM_003631.2	2581-2680	GTGCTGGATCACAATGAATGTCTAATTATCACAGGTACTGAGCAGTACAGTGAATACACAGGCTATGCTGAGACATATCGTTGGTCCCGGAGCCACGAAG
PGK1	NM_000291.2	1031-1130	GCAAGAAGTATGCTGAGGCTGTCACTCGGGCTAAGCAGATTGTGTGGAATGGTCCTGTGGGGGTATTTGAATGGGAAGCTTTTGCCCGGGGAACCAAAGC
PPIA	NM_021130.3	316-415	TCTATGGGGAGAAATTTGAAGATGAGAACTTCATCCTAAAGCATACGGGTCCTGGCATCTTGTCCATGGCAAATGCTGGACCCAACACAAATGGTTCCCA
PPP1CC	NM_002710.2	268-367	ACATCGACAGCATTATCCAACGGCTGCTGGAAGTGAGAGGGTCCAAGCCTGGTAAGAATGTCCAGCTTCAGGAGAATGAAATCAGAGGACTGTGCTTAAA
PROX1	NM_002763.3	1921-2020	TCTCCTTGTCGCTCATAAAGTCCGAGTGCGGCGATCTTCAAGATATGTCTGAAATATCACCTTATTCGGGAAGTGCAATGCAGGAAGGATTGTCACCCAA
PRRX1	NM_022716.2	331-430	GAAGGAATAGGACAACCTTCAATAGCAGCCAGCTGCAGGCTTTGGAGCGTGTCTTTGAGCGGACACACTATCCTGATGCTTTTGTGCGAGAAGACCTTGC
RP11-247A12.2.1	ENST00000372490.1	428-527	CTCCCCATCACCGCACTCCCCATCGTGGCACTTCCCTTGTTGCAGTTTTATGGAGTGTGCGTCTGGCTCTCCAACTAGACTTGAACCGCTTGAGTGCATA
RPL19	NM_000981.3	316-415	CCAATGCCCGAATGCCAGAGAAGGTCACATGGATGAGGAGAATGAGGATTTTGCGCCGGCTGCTCAGAAGATACCGTGAATCTAAGAAGATCGATCGCCA
SVEP1	NM_153366.3	5726-5825	AATGGCCACTCCTCAGGTGAGATTTATACAGTAGGTGCCGAAGTCACATTTTCGTGTCAGGAAGGATACCAGTTGATGGGAGTAACCAAAATCACATGTT
TBP	NM_001172085.1	588-687	ACAGTGAATCTTGGTTGTAAACTTGACCTAAAGACCATTGCACTTCGTGCCCGAAACGCCGAATATAATCCCAAGCGGTTTGCTGCGGTAATCATGAGGA
TFEC	NM_012252.2	891-990	CTTCACTTGGCACGGTTGATTTAGGTGCTCATGTCACCAAACAGCAGAGCCATCCTGAGCAGAATTCAGTAGACTATTGCCAACAACTGACTGTGTCTCA
TGFBR1	NM_004612.2	1256-1355	GAATCCTTCAAACGTGCTGACATCTATGCAATGGGCTTAGTATTCTGGGAAATTGCTCGACGATGTTCCATTGGTGGAATTCATGAAGATTACCAACTGC
THBS1	NM_003246.2	3466-3565	ACCCTCGTCACATAGGCTGGAAAGATTTCACCGCCTACAGATGGCGTCTCAGCCACAGGCCAAAGACGGGTTTCATTAGAGTGGTGATGTATGAAGGGAA
THBS2	NM_003247.2	4461-4560	AAACATCCTTGCAAATGGGTGTGACGCGGTTCCAGATGTGGATTTGGCAAAACCTCATTTAAGTAAAAGGTTAGCAGAGCAAAGTGCGGTGCTTTAGCTG

ZNF444	NM_001253792.1	1439-1538	CCACTTGGCCTCTTCCTCTCCTCCTTCCCTCCCATCGTCCTCCTCCACCTGCGCCTCCCTTGTCTGAACTTCCCAACGCCTTCCTATTCCTTTCCAACTC

Table S2. Amplification primers used for ddPCR and qPCR analysis

Gene	Primer	Length
GAPDH	Fw- 5’-GCA CCG TCAA GGC TGA GAA C-3’	96
	Rev- 5’-CAT CGC CCC ACT TGA TTT TG-3’	
ACTB	Fw -5’-GTG GAT CAG CAA GCA GGA GT-3’	95
	Rev 5’-AGG GTG TAA CGC AAC TAA GTC-3’	
AKT1	Fw 5’-CCA CTG TCA TCG AAC GCA CCT-3’	77
	Rev 5’-CAC AGT CTG GAT GGC GGT TGT-3’	
AKT2	Fw 5’-AAG CAG AGG CTT GGT GGG-3’	71
	Rev 5’-TTG ATG CTG AGG AAG AAC CTG-3’	
AKT3	Fw 5’-TCG AGA GAG CGG GTG TTC T-3’	77
	Rev 5’-TGT AGA TAG TCC AAG GCA GAG A-3’	
KLF16	Fw 5’-GTC AGA CCC AGA AAG GTG GA-3’	78
	Rev 5’-GGA CGG TTC TGG AAC AAA AG-3’	
HPRT1	Fw 5’-GTG TCA TTA GTG AAA AGC A-3’	91
	Rev 5’-CGA TGT CAA TAG GAC TCC AGA TGT T-3’	

Figure S1 Agilent BioAnalyzer profiles of matched FFPE/BFPE pairs and respective RIN numbers.

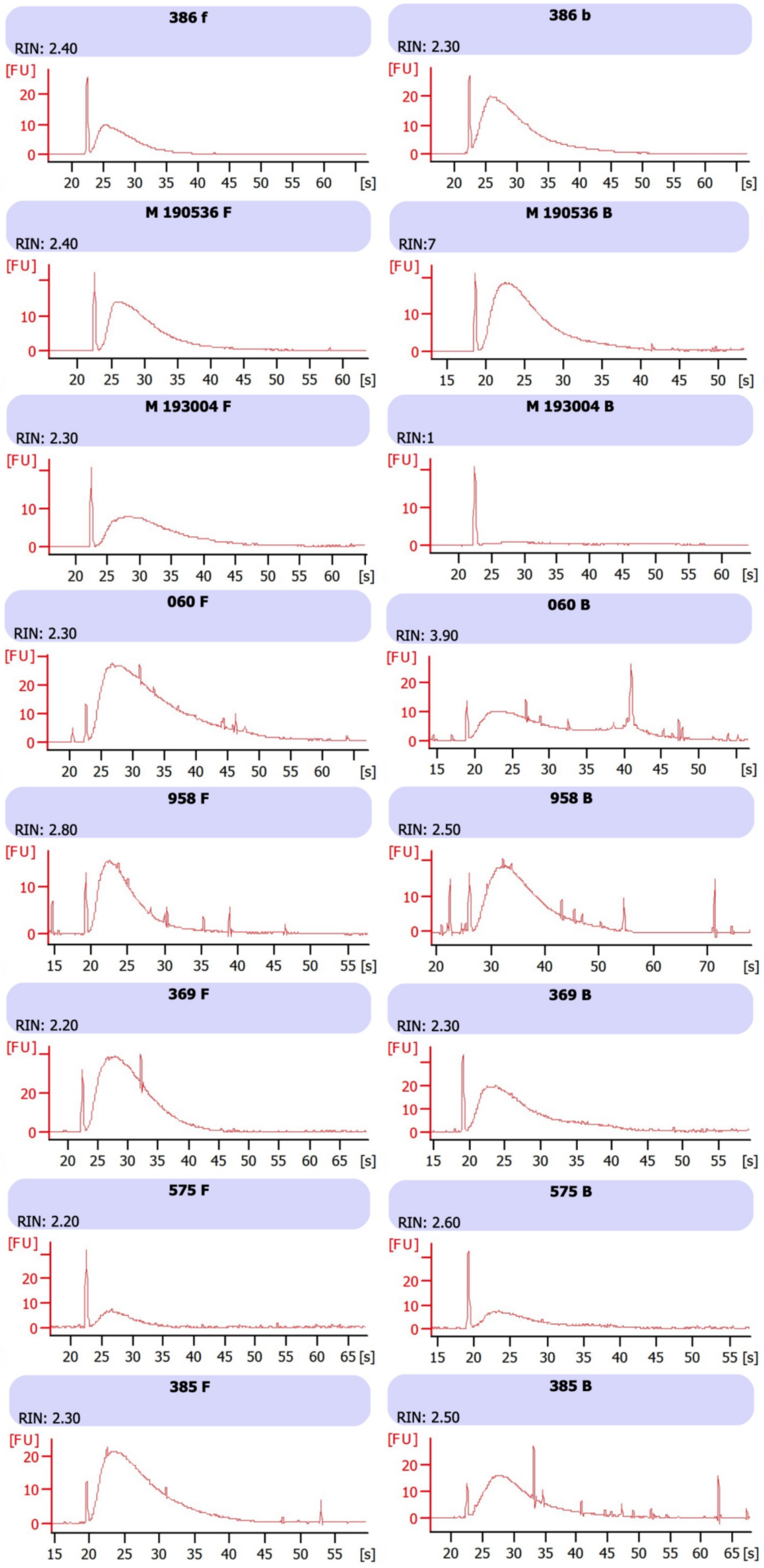

Figure S2 Results of the isolation procedure in the eight matched formalin/Bouin's samples. (A) RNA yield after isolation. (B) RNA Integrity Number (RIN). (C) Percentage of RNA fragments according to different smear regions as measured by smear analysis on Agilent BioAnalyzer. (D, E) RNA purity as measured by A260/280 and A260/230 ratios on Nanodrop.

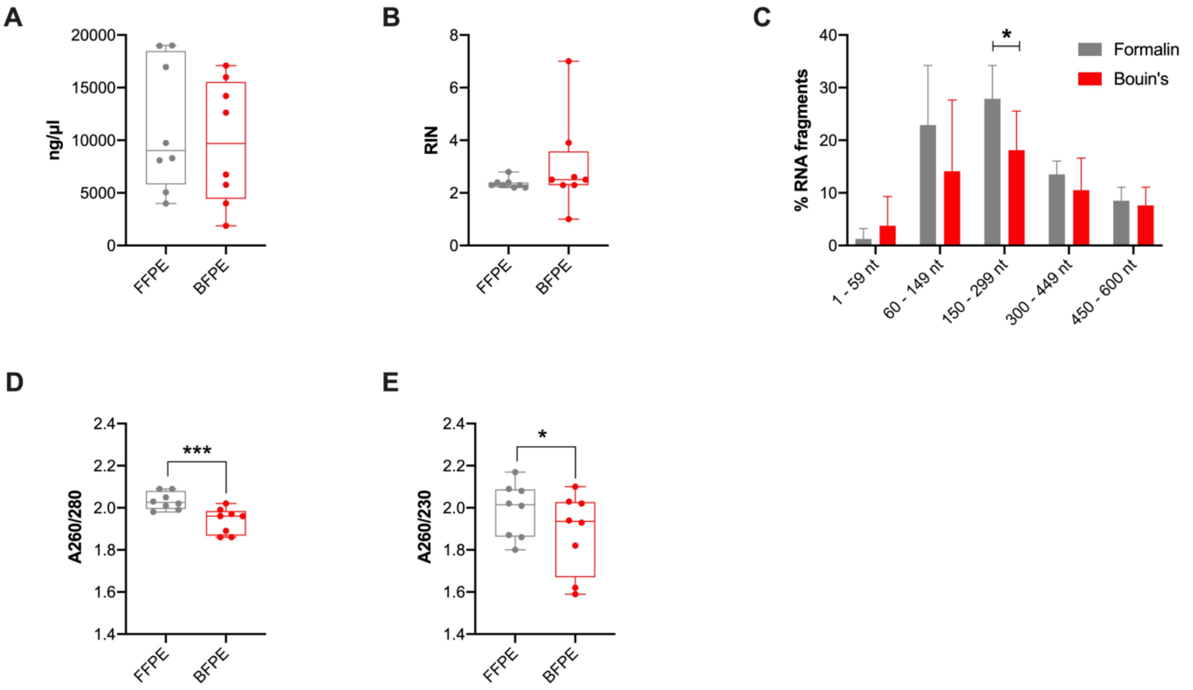

Figure S3 Volcano plot for differentially expressed genes on matched formalin and Bouin's tissues identified on Nanostring, ddPCR and qPCR using raw data (C).

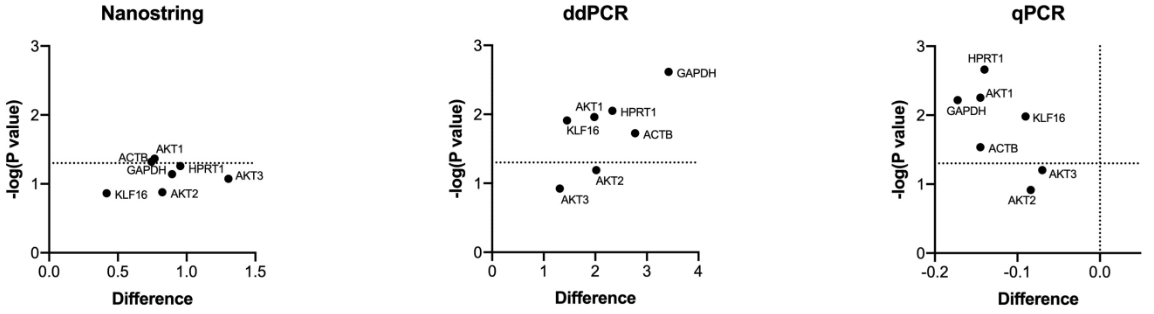

Supplement: Supplementary file 1 [file mps-07-00040-s001.zip › mps-2944272-supplementary.pdf]
